# Hexagonal gradient scheme with RF spoiling improves spoiling performance for high‐flip‐angle fast gradient echo imaging

**DOI:** 10.1002/mrm.26213

**Published:** 2016-04-01

**Authors:** Aaron T. Hess, Matthew D. Robson

**Affiliations:** ^1^ University of Oxford Centre for Clinical Magnetic Resonance Research, Division of Cardiovascular Medicine, Radcliffe Department of MedicineOxfordU.K.

**Keywords:** fast gradient echo, spoiling, RF spoiling, gradient spoiling

## Abstract

**Purpose:**

To present a framework in which time‐varying gradients are applied with RF spoiling to reduce unwanted signal, particularly at high flip angles.

**Methods:**

A time‐varying gradient spoiler scheme compatible with RF spoiling is defined, in which spoiler gradients cycle through the vertices of a hexagon, which we call hexagonal spoiling. The method is compared with a traditional constant spoiling gradient both in the transition to and in the steady state. Extended phase graph (EPG) simulations, phantom acquisitions, and in vivo images were used to assess the method.

**Results:**

Simulations, phantom and in vivo experiments showed that unwanted signal was markedly reduced by employing hexagonal spoiling, both in the transition to and in the steady state. For adipose tissue at 1.5 Tesla, the unwanted signal in the steady state with a 60 ° flip angle was reduced from 22% with constant spoiling to 2% with hexagonal spoiling.

**Conclusions:**

A time‐varying gradient spoiler scheme that works with RF spoiling, called “hexagonal spoiling,” has been presented and found to offer improved spoiling over the traditional constant spoiling gradient. Magn Reson Med 77:1231–1237, 2017. © 2016 The Authors Magnetic Resonance in Medicine published by Wiley Periodicals, Inc. on behalf of International Society for Magnetic Resonance in Medicine. This is an open access article under the terms of the Creative Commons Attribution License, which permits use, distribution and reproduction in any medium, provided the original work is properly cited.

## INTRODUCTION

Incomplete spoiling of transverse magnetization in spoiled gradient‐refocused echo (GRE) sequences with large flip angles (those significantly larger than the Ernst angle) results in large signal variations during the approach to the steady state 1. These may present as visible artifacts in fast imaging, such as prospectively gated cardiac cine. Incomplete spoiling can also reduce the accuracy of quantitative methods based on multiple measurements with a variable flip angle such as those used for T_1_ and B_1_ mapping 2, 3. These signal fluctuations manifest as artifacts in the phase‐encoding direction, potentially rendering images unusable. Crawley et al 4 introduced the concept of using spoiler gradients to reduce the visibility of the refocused and unwanted signal. Zur et al 5 introduced radio frequency (RF) spoiling, in which the phase of successive RF pulses increments quadratically, rendering refocused echoes incoherent with respect to each other. However, Epstein et al 1 commented that when imaging with high flip angles and/or a long T_2_, RF spoiling produces “jagged variations” in the signal evolution.

In the RF spoiling work by Zur et al 5, a requirement was established that the gradient moment accumulated in each repetition period should not change, and thus results in a constant spoiler gradient. In this work we describe how to relax this condition and demonstrate a gradient spoiling scheme with time‐varying spoiler gradients. From this a six‐point, three‐dimensional (3D), time‐varying spoiler gradient scheme, called “hexagonal spoiling,” is evaluated. We hypothesize that using higher‐order gradient spoiling schemes will enhance the natural spoiling effects of T_1_ and T_2_ relaxation and diffusion by increasing the number of pulse repetition time (TR) periods between the initial excitation and the refocusing of this unwanted signal. Hexagonal spoiling is compared with constant gradient spoiling both with and without RF spoiling using simulations and phantom experiments. In vivo spoiled GRE images were acquired to evaluate the new time‐varying spoiler scheme with RF spoiling.

## THEORY

In this section, extended phase graph (EPG) 6, 7, 8 theory is employed to describe the requirements for RF and gradient spoiling. EPG theory represents magnetization as discrete Fourier components that are manipulated by RF pulses and gradients using the operators  T(α,Φ) and S(Δk), respectively. The effect of an RF pulse of flip angle α and phase Φ is

(1) $${\left[\begin{array}{c} {\tilde{F}}_{k}\\ {\tilde{F}}^{*}{}_{-k}\\ {\tilde{Z}}_{k} \end{array}\right]}^{+}=T(\alpha ,\Phi ){\left[\begin{array}{c} {\tilde{F}}_{k}\\ {\tilde{F}}^{*}{}_{-k}\\ {\tilde{Z}}_{k} \end{array}\right]}^{-}\text{where},\;T(\alpha ,\Phi )=\;[\begin{array}{ccc} coS^{2}(\frac{\alpha }{2}) & e^{2i\Phi }\sin^{2}(\frac{\alpha }{2}) & -ie^{i\Phi }\sin(\alpha )\\ e^{-2i\Phi }\sin^{2}(\frac{\alpha }{2}) & \cos^{2}(\frac{\alpha }{2}) & ie^{-i\Phi }\sin(\alpha )\\ -\frac{i}{2}e^{-i\Phi }\sin(\alpha ) & \frac{i}{2}e^{i\Phi }\sin(\alpha ) & cos(\alpha ) \end{array}].$$

In Eq. (1), the superscripts − and + depict magnetization before and after an RF pulse. ${\tilde{F}}_{k}$ is the transverse magnetization for Fourier component k, in which ${\tilde{F}}_{k}^{*}$ is the complex conjugate, and ${\tilde{Z}}_{k}$ is the longitudinal magnetization with Fourier component k. k, indicating a discrete Fourier component, can be one, two, or three dimensional. A gradient with moment Δk leads to

(2) $$S\left(\Delta k\right):\;{\tilde{F}}_{k}\;\to {\tilde{F}}_{k+\Delta k}\;\mathrm{and}\;{\tilde{Z}}_{k}\to {\tilde{Z}}_{k}$$

where S(Δk) is a simple shift operator applied only to the transverse magnetization.

**Figure 1:**
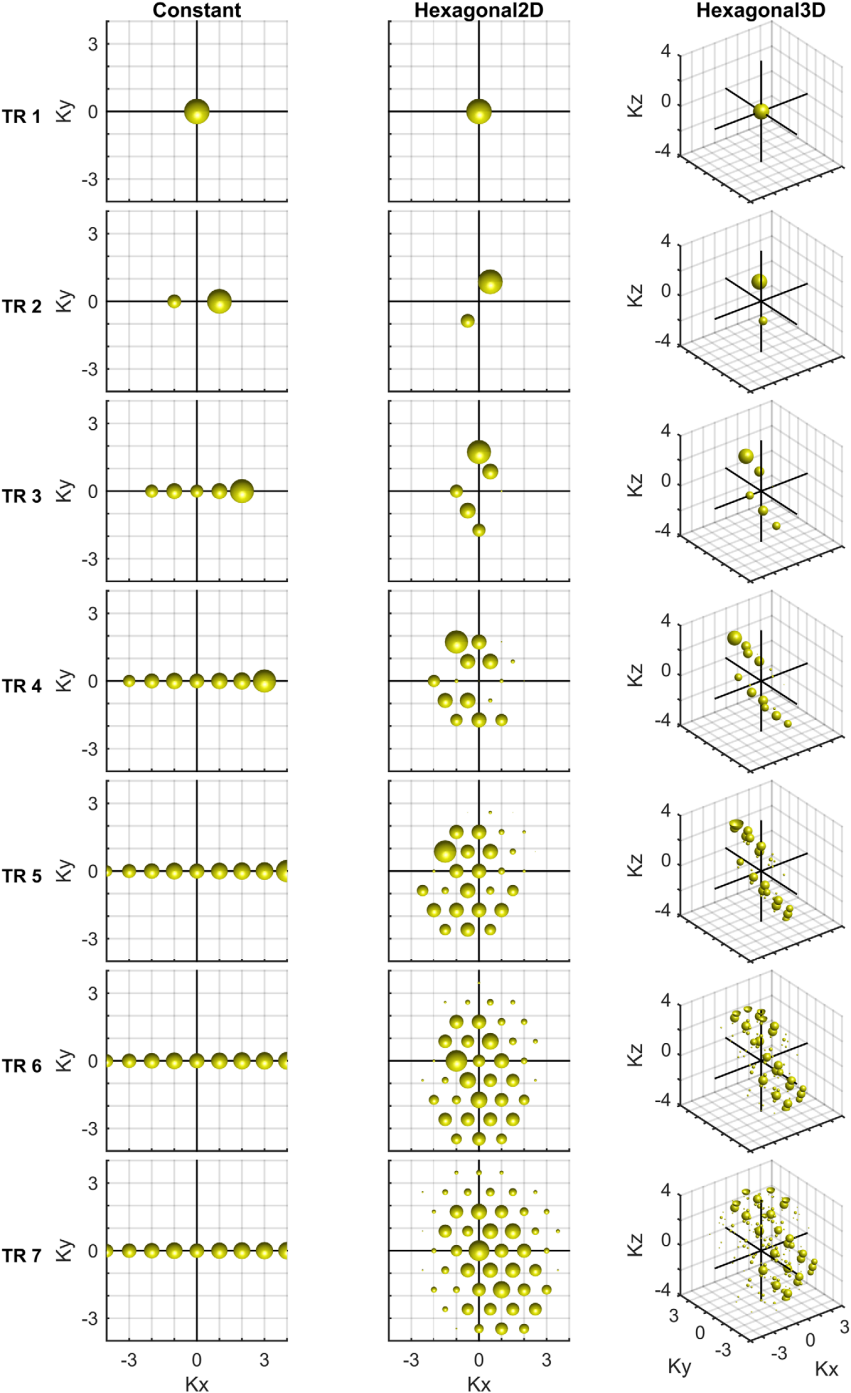
Fourier components generated from the first FID in a train of 20 ° RF pulses for three gradient spoiling schemes. The spheres' radii are proportional to the log of transverse magnetization. Only the transverse magnetization from the first RF pulse is considered; all other fresh magnetization is not shown. T_1_ and T_2_ were assumed to be infinitely large. The three gradient spoiling schemes are a constant spoiler gradient in Kx, a 2D hexagon in Kx and Ky, and a hexagon with a constant spoiler in the third dimension, Kz. One axis unit corresponds to the magnitude of a single spoiler gradient. [Color figure can be viewed in the online issue, which is available at wileyonlinelibrary.com.]

The form of the operator T(α,Φ) demonstrates that with every RF pulse, magnetization is shifted only between ${\tilde{F}}_{k}$, ${\tilde{F}}^{*}{}_{-k}$, and ${\tilde{Z}}_{k}$ for each k. RF spoiling results from the mixing of these states by an RF pulse when each of these states has experienced a different history of Φ, leading to a reduction in unwanted signal by phase cancellation. For efficient spoiling, any applied spoiling gradient, S(Δk), must shift all ${\tilde{F}}_{k}$ to arrive at a new k that already contains magnetization from previous RF pulses with differing phases. Traditionally, this is ensured by using a constant spoiler gradient 5. Figure 1 column 1 shows the buildup of magnetization in Fourier space with constant (*k*_*x*_) gradient spoiling. The first spoiler gradient shifts ${\tilde{F}}_{0}$ to ${\tilde{F}}_{1}$, outside the field of view. The second RF pulse shifts magnetization into ${\tilde{F}}^{*}{}_{-1}$, which is then refocused by the second spoiler gradient. To reduce this unwanted signal RF, spoiling is employed, although with such a short configuration history, it is unlikely to be complete. To prevent this refocusing, a time‐varying gradient regime can be considered in which the gradient moment is doubled every repetition, but this rapidly leads to impractically large spoiler gradients 9.

We now generalize the constant spoiling of Zur et al 5 to multiple dimensions of k.

Consider a set of spoiler gradient moments, $M_{i}^{\prime }$, defined as the addition or subtraction of the three primary spoiling moment vectors ( $M_{1}$, $\;M_{2}$, $\;M_{3}$) as follows:

(3) $$M_{i}^{\prime }=a_{i}M_{1}+b_{i}M_{2}+c_{i}M_{3}$$

where, $a_{i}$, $b_{i}$, and $c_{i}$ are integers. The consequence of applying these spoiling gradients is that all coherences can only lie on points defined by

(4) $${\tilde{k}}^{\prime }=xM_{1}+yM_{2}+zM_{3}$$

where x,y, and z are drawn from the set of integers *z*.

If one selects $M_{1}$ to $M_{3}$, so that they span 3D space, then Eq. (4) provides the definition of a Bravais Lattice. This definition enforces translational invariance in which the neighborhood of possible points around any single ${\tilde{k}}^{\prime }$ is identical to every other. Such sets of gradient spoiler moments fulfill the first requirement for spoiling gradients: different coherence pathways result in the same ${\tilde{k}}^{\prime }$, leading to signal cancellation by RF spoiling 5.

The second requirement for spoiling gradients is that their moment should be greater than the maximum spatial frequency of interest. This is usually the extreme of the sampled image's k‐space, $k_{image}$. Thus, magnetization with $\left|k_{x}\right|>\left|2k_{x,image}\right|$ or $\left|k_{y}\right|>\left|2k_{y,image}\right|$ is considered spoiled. For this reason we can set a constant amplitude $\left|M_{i}^{\prime }\right|>\left|2k_{x,image}\right|$.

In two dimensions (2D), there are only two schemes in which $\left|M_{i}^{\prime }\right|$ is constant. In the first scheme, which corresponds to square symmetry, (a_i_,b_i_) must be drawn from (1,0), (0,1), (−1,0) or (0,−1). In the second case of hexagonal symmetry, the angle between $M_{1}^{hex}$ and $M_{2}^{hex}$ is 60 ° and (a_i_,b_i_) can be any of (1,0), (0,1), (−1,1), (−1,0), (0,−1), and (1,−1).

The hexagonal case is the scheme that has been evaluated in this work for two reasons. First, it has rotational symmetry about the center of k‐space, which ensures a constant cumulative effect of all preceding and forthcoming gradients, no matter which RF pulse the magnetization originates from. Second, the hexagon has a higher number of spoiling gradient combinations than the square case, leading to a longer duration before states are refocused.

Iterating around the vertices of a hexagon in 2D delays the refocusing of the first unwanted signal until the fourth repetition. This Fourier component buildup is shown in Figure 1 column 2, although the first unwanted signal in the fourth repetition is too small to plot.

The benefit of applying the third dimension of spoiling is to further delay the refocusing of the magnetization, and hence to allow it further attenuation through T_1_ and T_2_ relaxation and diffusion. In 3D, once again we can apply the standard spoiling moment requirement in which $\left|M_{i}^{\prime }\right|=constant>\left|2k_{x,image}\right|\;$. Although there are multiple schemes for selecting $M_{1}$, $M_{2}$, and $M_{3}$ owing to the increased dimensionality of the system, the hexagonal arrangement was again chosen for its large number of vertices and symmetry. We can follow from the hexagonal spoiling approach described in 2D, but in this case using the additional gradient moment applied perpendicular to the plane of $M_{1}^{hex}$ and $M_{2}^{hex}$, and ( $a_{i}$, $b_{i}$, $c_{i}$) in the sequence (1,0,1), (0,1,1), (−1,1,1), (−1,0,1), (0,−1,1), and (1,−1,1). Figure 1 column 3 shows the buildup of coherences with this method that further delays the refocusing of the first unwanted coherence until the sixth RF pulse, which is too small to be observed in Figure 1. This 3D hexagonal spoiling method is evaluated in the remainder of this work.

## METHODS

EPG simulations were implemented in MATLAB (The Mathworks, Natick, Massachusetts) following the methodology of Weigel 6. The simulations were performed with discrete 3D Fourier component bins ( k), separated in $k_{x}$ by cos (60 °), in $k_{y}$ by sin (60 °) and in $k_{z}$ by 1. EPG simulations were set up to compare a constant spoiling gradient to (3D) hexagonal spoiling using a TR of 10 ms and 60 repetitions for all cases. T_1_ and T_2_ were chosen to represent that of peanut oil, because of its close resemblance to adipose tissue 10. Inadequate spoiling of adipose tissue can cause substantial artifacts because of its long T_2_ relative to its T_1_, low diffusivity, and because it appears subcutaneously in regions of high RF‐coil sensitivity. The T_1_ and T_2_ values of peanut oil were quantified on a 1.5 T scanner (Avanto, Siemens, Munich, Germany) by fitting the main spectral peak observed in stimulated echo and spin echo spectroscopic measurements to the expected signal. T_1_ and T_2_ were found to be 180 and 65 ms, respectively. Unless otherwise stated, an RF spoiling phase of 50 ° was used in the simulations.

Five different simulations were run:

1. T_1_s of 180, 434, and 300–1500 ms in 100‐ms steps, T_2_ = TR/10 (giving ideal spoiling), flip angles of 1 ° to 90 ° in steps of 1 °.
2. T_1_s of 300 to 1500 ms in 100‐ms steps, T_2_s of 10 to 250 ms in 10‐ms steps, flip angles set from 5 ° to 90 ° in 5 ° steps for both constant and hexagonal spoiling with RF spoiling.
3. T_1_ of 180 ms, T_2_ of 65 ms (peanut oil at 1.5 T), flip angles of 1 ° to 20 ° in steps of 1 °, and 20 ° to 90 ° in steps of 5 °, both with and without RF spoiling, for both constant and hexagonal spoiling gradients.
4. T_1_ of 180 ms, T_2_ of 65 ms (peanut oil at 1.5 T), flip angle of 60 °, four RF spoiling phases, 0 ° (none), 50 °, 117 °, and 123 ° for both constant and hexagonal spoiling gradients.
5. T_1_ of 434 ms, T_2_ of 43 ms (peanut oil at 7 T 11), flip angle of 60 ° for both constant and hexagonal spoiler gradients with RF spoiling.

The 3D hexagonal spoiling scheme was implemented in a spoiled GRE pulse sequence in which the time‐varying gradients were assigned to the readout and slice select axes, and the constant ( $M_{3}$) gradient was assigned to the phase encode axis. This choice was informed by an empirical assessment of image quality and likely relates to the eddy current effects. Imaging gradient moments were accounted for when calculating the spoiler moments. Phantom experiments were carried out on a 1.5T scanner (Avanto, Siemens) and human 7T scanner with constant spoiling and hexagonal spoiling, with RF spoiling phase of 50 °. A peanut oil phantom measuring 35 × 35 × 60 mm^3^ was used.

To study the approach to steady state, in each 1.5T phantom experiment 176 RF pulses were applied with a TR/echo time (TE) of 10 ms/2 ms at flip angles of 1 ° to 20 ° in 1 ° increments, and 20 ° to 90 ° in 5 ° increments, readout bandwidth of 300 Hz/pixel with a 12‐channel head coil. A nonselective RF pulse was used to avoid slice profile effects, with a readout field of view (FOV) of 256 mm with 176 readout points and no phase‐encoding gradients. At 7 T, a transceiver birdcage head coil (375‐mm diameter), 512 RF pulses, TR/TE of 10 ms/2 ms and a slab selective 60 ° 250 mm excitation, were used. The mean signal of each readout was used to study the signal variation in the first 30 TR periods and the final steady‐state signal amplitude. These were compared with the theoretical spoiled steady state, $S_{ss}$, and signal in the approach to the steady state for a spoiled gradient echo sequence S(j):

(5) $$S_{ss}=c_{scale}\frac{\sin(\alpha )(1-e^{-\frac{TR}{T1}})}{\left(1-cos(\alpha )e^{-TR/T1}\right)}$$

(6) $$S(j)=c_{scale}\sin\left(\alpha \right)[f_{z,ss}+{\left(\cos\left(\alpha \right)e^{-\frac{TR}{T1}}\right)}^{j-1}\left(1-f_{z,ss}\right)]$$

where α is the flip angle, j is the RF pulse counter, and the variable $f_{z,ss}$ is the steady state of the longitudinal magnetization for a spoiled acquisition 12. The scaling constant $c_{scale}$ was calculated using a least squares fit to the measured $S_{ss}$ from the lowest five flip angles of the two RF‐spoiled measurements.

Unwanted signal is calculated for each TR number *j* as the relative difference between the measured signal and the ideal simulation or the theoretical signal equation S(j):

(7) $$unwanted\;signal\left(j\right)=\frac{S_{m}\left(j\right)-S(j)}{S(j)}$$

where $S_{m}(j)$ is the refocused transverse magnetization from method m. Perfect spoiling would yield unwanted signal = 0.

The method was evaluated on a healthy volunteer at 1.5 T with a transverse‐spoiled GRE acquisition through the liver with matrix of 144 × 101, FOV of 380 × 380 mm, flip angles of 1 °, and 5–90 ° in steps of 5 °, TR/TE of 5/3 ms, and bandwidth of 300 Hz/pixel. Images were acquired with a constant spoiling gradient in the phase encode and readout equal to 1.2 times the maximum spatial frequency in the readout direction, and hexagonal spoiling in which $M_{1}^{hex}$ to $M_{3}^{hex}$ were again 1.2 times the maximum spatial frequency in the readout direction. RF spoiling was used in both acquisitions. This scan was acquired in accordance with our institution's ethical practices. The severity of artifacts was quantified in the background, when only noise is expected, by calculating the root mean square (RMS) of background intensity, normalized by the same region in a noise‐only acquisition.

## RESULTS

**Figure 2:**
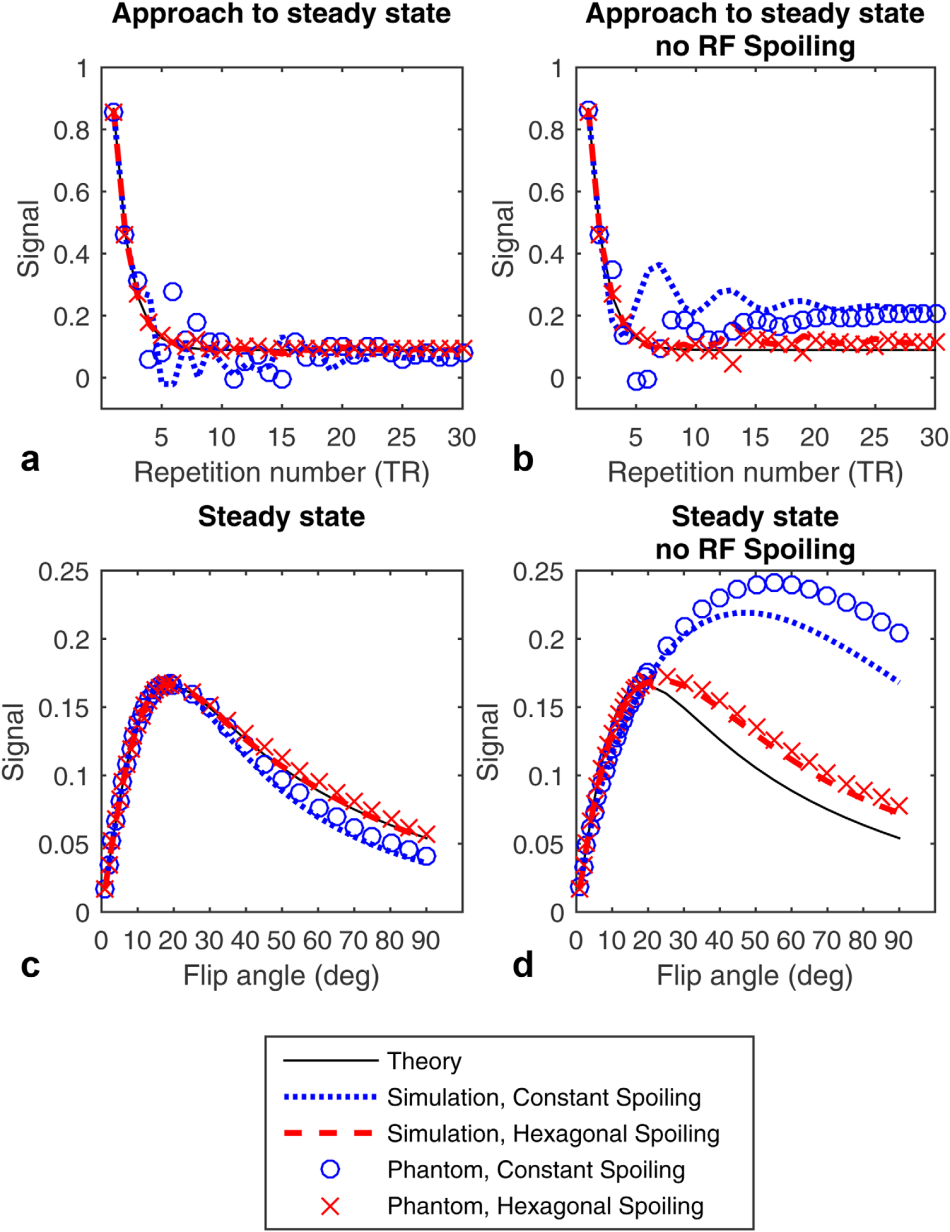
Plots of the real part of the signal amplitude expected from textbook theoretical equations, simulation, and phantom measurement. The plots shown are for a 60 ° excitation; the steady state in simulation is the 60th repletion and in measurement the 176th. Approach to the steady state with and without RF spoiling, respectively (a and b); steady state with and without RF spoiling, respectively (c and d).

Figure 2 shows the simulations and phantom measurements at 1.5 T in the approach to the steady state for a flip angle of 60 °, and the steady state reached as a function of excitation flip angle. The plot shows that with constant spoiling, large deviations from perfectly spoiled theory are observed that are reduced by hexagonal spoiling. The phantom and simulations demonstrate the same general characteristics, but the fine details differ.

The unwanted signal and phase calculated from simulation over the first 60 TRs for both spoiling regimes are shown in Supporting Figure S1 for flip angles of 30 °, 60 ° and 90 °. The mean and standard deviation of unwanted signal over the first 30 repetitions is plotted with T_1_ against T_2_ at a 60 ° flip angle, and T_2_ against flip angle at a T_1_ of 1 s in the Supporting Figures S2 and S3, respectively, that demonstrate improvements in spoiling using the hexagonal approach across the entire range of T_1_, T_2_, and flip angle space.

Table 1 presents the unwanted signal both while transitioning to, and in, the steady state calculated from both simulation and phantom experiments at 1.5 T and 7 T with a 60 ° flip angle, quantifying the unwanted signal seen in Figure 2 and Supporting Figure S1. The table further lists the simulations of three different RF spoiling phases, demonstrating that 50 ° is a reasonable increment. 50 ° was then used in the phantom and in vivo experiments. In all cases the standard deviation in the approach to the steady state was reduced with hexagonal spoiling. Likewise, the steady‐state unwanted signal was reduced when using hexagonal spoiling for all cases, showing that it brought the steady state closer to the theoretical, ideal steady state.

**Table 1:** Unwanted Signal, Mean, and Standard Deviation in First 30 Repetitions, and at Repetition 60 for Simulations and 176 for Phantom to Represent the Steady State^a^

			Transition to steady‐state unwanted signal (Mean ± SD) (%)		Steady‐state unwanted signal (%)	
Method	Field	RF spoiling phase	Constant	3D hexagonal	Constant	3D hexagonal
S imulation	1.5 T	123 °	62 ± 66	8 ± 11	30	4
		117 °	58 ± 61	8 ± 11	32	4
		50 °	55 ± 64	5 ± 7	22	2
		0 °	147 ± 72	24 ± 23	139	26
Phantom	1.5 T	50 °	62 ± 70	11 ± 8	38	12
		0 °	91 ± 68	23 ± 19	170	34
Simulation	7 T	50 °	40 ± 130	0 ± 20	20	2
Phantom	7 T	50 °	170 ± 140	14 ± 10	42	9

a All results reported from 60 ° flip angle experiments and T_1_ and T_2_ of peanut oil and a peanut oil phantom.

**Figure 3:**
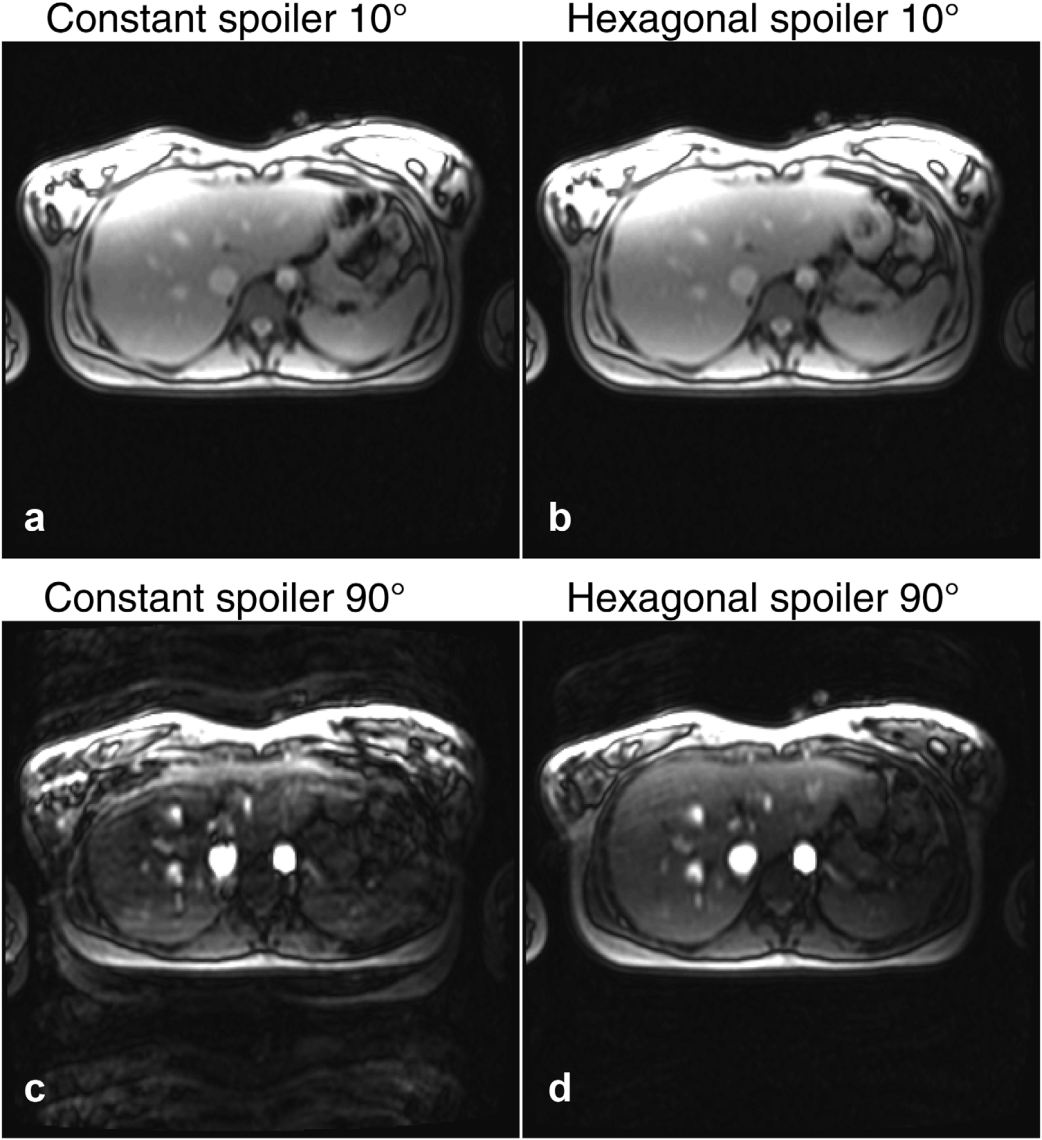
In vivo spoiled gradient echo images acquired with 101 readout lines during a breath hold. (a and c) Acquired with a constant spoiler at flip angles of 10 ° and 90 °. The artifacts in the 90 ° excitation overwhelm the image of the liver. (b and d) The same images acquired using hexagonal spoiling with identical spoiler magnitude.

**Figure 4:**
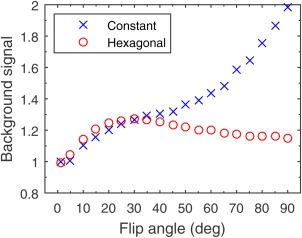
RMS background intensity of in vivo liver spoiled‐gradient‐echo images for flip angles between 1 ° and 90 °, normalized to the same measure in a noise reference image. [Color figure can be viewed in the online issue, which is available at wileyonlinelibrary.com.]

Figure 3 presents four images from a volunteer at 1.5 T, two with constant spoiling and two with hexagonal spoiling, at 10 ° and 90 ° excitation flip angles. Figure 4 plots the RMS background intensity as a function of flip angle, normalized to that of a noise reference scan. The figure shows an increase in background artifacts with a constant spoiler as the flip angle increases; for hexagonal spoiling this artifact is reduced above 30 °.

## DISCUSSION

This paper presents a scheme of time‐varying spoiler gradients that work in conjunction with RF spoiling. A six‐point spoiler gradient regime was defined around the vertices of a hexagon with two time‐varying gradients and one constant gradient. This scheme substantially reduces the relative amplitude of unwanted signal at high flip angles.

Both phantom measurements and simulations demonstrated good alignment to the theoretical or ideal transition to—and steady state for—hexagonal spoiling, as shown in Figure 2. When using RF spoiling with a constant spoiling gradient, the transition to the steady state is marked by large oscillations in the magnitude and phase of the received signal. There is some disagreement between the phantom measurements and simulation, particularly in this approach to steady state, which may be the result of small differences between simulated and executed RF phase increment. These oscillations are most evident at flip angles over 30 °, and for almost all T_1_ and T_2_s simulated, with the exception of very short T_2_s (T_2_ ∼ TR).

The unwanted signal in the steady state in phantom and simulation was again slightly different, although it was consistently reduced with hexagonal spoiling, as given in Table 1. The largest change was in a phantom at 7 T using a 60 ° flip angle; the relative amplitude of unwanted signal in the steady state with a constant spoiler was as high as 42%, whereas hexagonal spoiling reduced this to 9%. Differences of this magnitude would be significant for quantitative methods that measure tissue and $B_{1}^{+}$ characteristics under the assumption of ideal spoiling.

Other methods have been proposed to minimize the effect of unwanted coherences in the approach to steady state. One such method is to use a prepulse and wait period to get the longitudinal magnetization into its steady state 13. This method requires previous knowledge of flip angle and T_1_ and for them to be within a small range. Other methods have been presented that reduce unwanted coherences by averaging or filtering multiple RF‐spoiled acquisitions with different RF spoiling cycles 14, 15. Related to this is the use of random spoiler gradients in a radial acquisition, in which the unwanted coherences effectively cancel each other out in the oversampled central k‐space 16.

The limitation of time‐varying spoiler gradients is that eddy currents will also vary between repetitions. Their manifestation will depend on both the performance of the scanner's eddy current compensation and the orientation of the applied gradients. Eddy current artifacts caused by hexagonal spoiling are likely to manifest as repeats of the image along the phase‐encode direction, as a result of the cyclic nature of the scheme. Figures 3 and 4 demonstrate that the benefits from the method can outweigh these effects, but eddy current effects may limit this method on some MRI hardware. A further limitation of time‐varying spoiler gradients is that there is a small time penalty in repetition time when compared with constant spoiling: The residual moment at the end of a Cartesian readout must be considered in the time‐varying gradient calculation.

There are several applications in which using this high‐flip‐angle spoiled GRE acquisitions method could be applied, and in which the artifacts seen in vivo (Fig. 3c) would render them unusable if a constant spoiler is used. The actual flip angle imaging (AFI) 17 $B_{1}^{+}$ mapping technique is an example of when two flip angles are interleaved, making the method sensitive to inadequate spoiling 2. Spoiled GRE T_1_ mapping with multiple flip angles is another method that is susceptible to inadequate spoiling 3. Other applications could include mitigating artifact when a large range of B_1_ is present, such as when using surface transmit coils, or when the inflow of blood causes nonsteady state behavior.

In conclusion, a time‐varying gradient spoiler scheme that works with RF spoiling, called hexagonal spoiling, has been presented and found to offer superior suppression of unwanted signal over the traditional constant spoiling gradient.

## Supporting information

Additional Supporting Information may be found in the online version of this article

**Fig. S1**. Simulations of the approach to the steady state for three flip angles: 30^°^, 60^°^, and 90^°^. (a and b) Unwanted signal calculated using Eq. 7. (c and d) Phase of the simulated signal (S_m_(j)) in Eq. 7, which is zero in the ideal case.
**Fig. S2**. Simulated plots of unwanted signal in the approach to steady state for a range of T_1_ and T_2_ values using a flip angle of 60^°^, demonstrating a consistent reduction in unwanted signal when using hexagonal spoiling.
**Fig. S3**. Simulated plots of unwanted signal in the approach to steady state for a range of flip angles and T_2_ values with T_1_ fixed at 1 s, demonstrating a consistent reduction in unwanted signal for flip angles over 30^°^.
