# Supplementary material for: Hexagonal gradient scheme with RF spoiling improves spoiling performance for high‐flip‐angle fast gradient echo imaging
Source: Magn Reson Med. 2016 Apr 1;77(3):1231–7. doi: 10.1002/mrm.26213 (PMC5324613; doi:10.1002/mrm.26213)
Supplement: Supplementary file 1 — Fig. S1. Simulations of the approach to the steady state for three flip angles: 30°, 60°, and 90°. (a and b) Unwanted signal calculated using Eq. 7. (c and d) Phase of the simulated signal (Sm(j)) in Eq. 7, which is zero in the ideal case. Fig. S2. Simulated plots of unwanted signal in the approach to steady state for a range of T1 and T2 values using a flip angle of 60°, demonstrating a consistent reduction in unwanted signal when using hexagonal spoiling. Fig. S3. Simulated plots of unwanted signal in the approach to steady state for a range of flip angles and T2 values with T1 fixed at 1 s, demonstrating a consistent reduction in unwanted signal for flip angles over 30°. [file MRM-77-1231-s001.docx]

**Supporting Figures**

Figure S1: Simulations of the approach to the steady state for three flip angles, 30°, 60°, and 90°. Plotted in a and b is the unwanted signal calculated as in equation 7. Plotted in c and d is the phase of the simulated signal (S_m_(j)) in eq. 7, which is 0 in the ideal simulation.

Figure S2: Plots of unwanted signal for a range of T1 and T2 values at a 60° flip angle, demonstrating a consistent reduction in unwanted signal when using hexagonal spoiling.

Figure S32: Plots of unwanted signal for a range of flip angles and T2 values, demonstrating a consistent reduction in unwanted signal for flip angles over 30°.
